# Non-canonical extracellular complement pathways and the complosome paradigm in cancer: a scoping review

**DOI:** 10.3389/fimmu.2025.1519465

**Published:** 2025-04-30

**Authors:** Camila de Freitas Oliveira-Tore, Amarilis Giaretta de Moraes, Helena Musetti B. S. Plácido, Nathalia M. D. L. Signorini, Pamela Dias Fontana, Tatiane da Piedade Batista Godoy, Angelica Beate Winter Boldt, Iara de Messias

**Affiliations:** ^1^ Laboratory Molecular Immunopathology, Postgraduate Program in Internal Medicine and Health Sciences, Federal University of Paraná (UFPR), Curitiba, PR, Brazil; ^2^ Laboratory Human Molecular Genetics, Postgraduate Program in Genetics, Department of Genetics, Federal University of Paraná (UFPR), Curitiba, PR, Brazil

**Keywords:** C3, C1q, C5, cancer, complement system, non-canonical activation pathway

## Abstract

The Complement System (CS) comprises three catalytic pathways that can be activated by specific immune triggers. However, within the tumor microenvironment (TME), CS intracellular components, recently named as complosome, play roles that extend beyond the activation and regulation of its pathways. The interaction between TME elements and tumor cells alters the local immune response, leading to inflammation, cell proliferation, and tumor invasion. Our focus is on understanding the significance of complosome and non-canonical pathways in cancer. In this scoping review, we analyzed 45 articles that discussed the various roles of CS components in carcinogenesis. Many CS components, including C1q, C3a-C3aR, C5a-C5aR, factor H, and properdin, some of them at the intracellular level, may play a dual role in tumor progression, demonstrating either anti-tumor or pro-tumor activity independent of complement pathway activation. The specific function of each component can influence both the type and stage of tumor cells. There is a notable lack of studies on the role of the lectin pathway in tumor development, and this knowledge gap must be addressed to fully understand the role of complosome in cancer. Nevertheless, the activation of CS and the roles of its components in complosome pathways are crucial steps in tumor development.

## Graphical Abstract

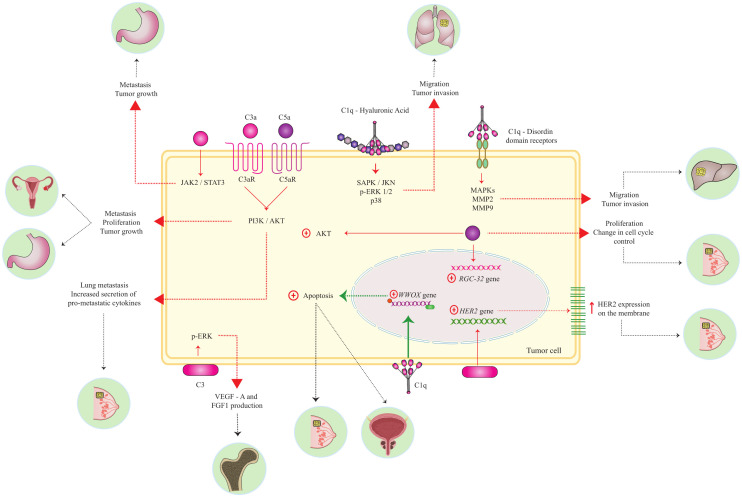

## Highlights

- C1q has an anti-tumor role through the *WWOX* oncogene in breast and colon cancers.
- The C3a-C3aR and C5a-C5aR axis activate the PI3K/AKT and ERK/MEK1-2 pathways.
- Activation of C3 plays a negative regulatory role for the oncogene Her2.
- C5a promotes overexpression of the *RGCC* gene, which controls cell cycle progression.
- Properdin showed an antitumor function by regulating pro-apoptotic factors.

## 1 Introduction

The complement system (CS) comprises a set of soluble, membrane-bound, and regulatory proteins that are involved in homeostasis processes. It serves as a central component of innate immunity while also providing a critical link to adaptive immunity. CS is known as a key mediator of inflammation, coagulation cascade, in the elimination of immune complexes and apoptotic cells (1–3).

**Figure 1:**
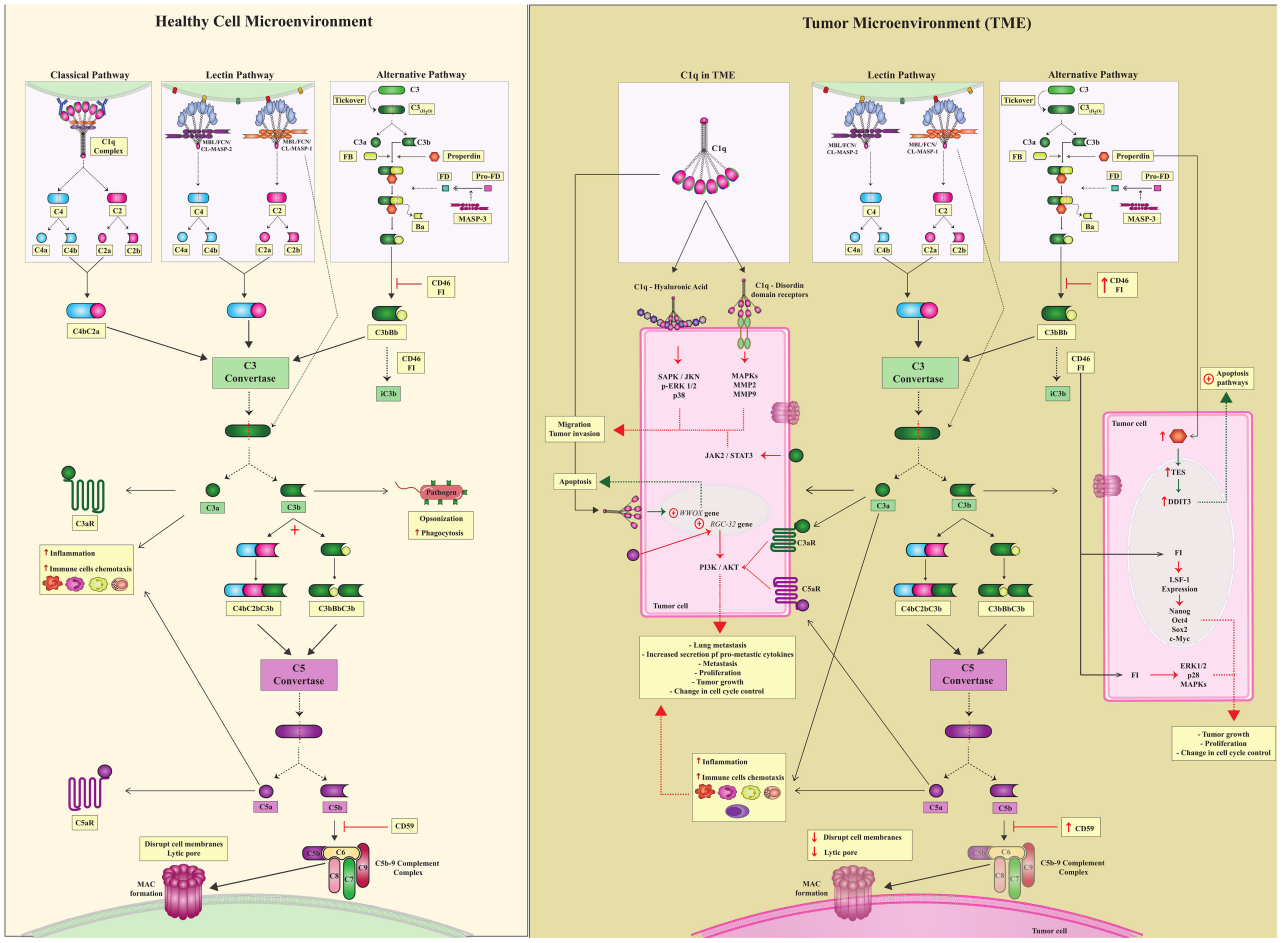
Canonical complement system and complosome pathways. (Healthy Cell Microenvironment) There are three canonical CS pathways in the microenvironment of healthy cells: classical, lectin, and alternative pathways. Different triggers activate all of them; however, these three pathways converge to form C3 convertase, which is a central complement enzyme. C3 converts cleaves C3 into C3a and C3b. C3a is a potent anaphylatoxin capable of attracting immune cells and contributing to inflammation. C3b continues in the complement cascade and forms another essential enzyme, C5 convertases. C5 convertase cleaves C5 into C5a and C5b.C5a, just as C3a is an anaphylatoxin, and C3b follows the cascade and leads to the formation of the C5bC9 complement complex, which culminates in the formation of MAC. To ensure controlled complement activation, complement regulators must act efficiently. (TME) In the tumor microenvironment, in addition to the canonical action of the complement system, there is also a complosome. Complosome proteins play a role in different intracellular pathways, leading to pro- or anti-tumor profiles in these cells. Tumor cells are pink. Green dotted arrows indicate the anti-tumor profile, and red dotted arrows indicate a pro-tumor profile.

Canonical complement activation induces proteolytic cleavage of key molecules through three pathways: the classical (CP), lectin (LP), and alternative (AP) pathways. CP activation occurs through antibody binding to the C1q-C1r/C1s complex (4, 5). LP is activated through the recognition of pathogen-associated, damage-associated, and altered cell-associated molecular patterns (PAMPs, DAMPs, and ACAMPs) by pattern recognition receptors (PRRs, as mannose-binding lectin -MBL, ficolins, and collectins), thereby activating the MBL-associated serine proteases MASP-1 or MASP-2 (mannan-binding lectin-associated serine proteases -1 or -2). Activation of the CP and LP pathways generate a crucial C3 convertase - C2aC4b. Yet the AP is continuously activated at low levels by the spontaneous hydrolysis of native C3, also circulating as C3(H_2_O), generating the C3bBb convertase (**Figure 1**). C3 convertases (C2aC4b or C3bBb) cleave C3 molecules into C3a and C3b fragments (6). The C3a fragment is an anaphylatoxin that may bind to its cell receptor, C3aR, influencing several immunological pathways (as discussed in this review), whereas C3b continues the cascade, leading to the formation of C5 convertase, which cleaves C5 into C5a and C5b. C5a is an effective chemotactic factor and proinflammatory mediator and like C3a, acts as an anaphylatoxin with several biological roles. C5b binds to C6, C7, C8, and C9 and forms the membrane attack complex (MAC), which may disrupt cell membranes by causing lytic pores (**Figure 1**) (5).

Recent evidence suggests that the role of the CS extends well beyond the three traditional catalytic pathways activated in the extracellular environment (7–10). To recognize this, two new terms have been introduced by complement researchers in the past decade: *complosome* and *non-canonical pathways* (NCP). They encompass the roles of complement proteins, regulators, and receptors that do not depend on activation of the canonical pathways CP, LP and AP. Although these concepts remain under discussion (9, 11), they will be used in this scoping review to emphasize the rather unexpected involvement of complement components in cancer development. Specifically, the term NCP will refer to complement activation within the extracellular environment, without the direct involvement of any of the three canonical pathways. In contrast, the term *complosome* will be used to describe the activity of intracellular complement components (**Figure 1**).

NCPs are less well defined but have gained increasing recognition for their roles in immune regulation and the modulation of inflammation. CS components that do not directly involve the activation of the CP, LP or AP in the extracellular space include serine proteases as MASPs, which interact with other proteins and receptors, such as protease-activated receptor 4 (PAR4) in endothelial cells (12–14), leading to pro-inflammatory and/or pro-thrombotic responses (15). They involve complex mechanisms, including activation via PRRs, tissue-specific factors, or indirect modulation through cytokine signaling, and contribute to the complement system’s broad regulatory functions in immunity, particularly in inflammation, tissue injury, and pathogen defense.

The complosome comprises all CS proteins able to act in intracellular processes independently of the activation of the canonical pathways. It represents a recent and evolving perspective, still requiring new study designs to achieve a more comprehensive definition (9, 11, 16, 17). Despite ongoing discussion regarding which pathways and functions of CS proteins can be specifically attributed to the complosome, it is evident that these proteins have the potential to regulate intracellular pathways through endosomes (18, 19), mitochondrial membranes (20, 21) and direct gene regulation (22, 23). The core complosome components are C3 and C5, as well as their respective membrane receptors. Furthermore, the complosome can be found in immune and non-immune cells, as well as in different tissues (11). Although still poorly investigated in the TME, the complosome may hold an additional key for a pro- or anti-tumor outcome (16).

Cancer is one of the most common health problems worldwide. In 2020, the International Agency for Research on Cancer reported 19.3 million new cases and almost 10 million deaths from cancer worldwide (24–26). The mechanisms underlying cancer development and progression are not completely understood. The TME is considered to be a key promoter of carcinogenesis, along with genetic and epigenetic factors (27–31). Crosstalk between TME elements and tumor cells modifies the local immune response, inducing inflammation and suppressing the anti-tumor response, thereby facilitating tumor proliferation and invasion (32–35). The hypothesis that chronic inflammation is closely related to malignant tumor transformation and progression has been substantiated by human epidemiological data and genetic experiments (36). However, the interplay between non-canonical complement pathways, the complosome and the TME has been largely out of the spotlight in cancer research. In this scoping review, we discuss the available data published till 2020 at the light of recent evidence, focusing on understanding the implications of activating these poorly known arms of the CS, within tumoral cells and the TME.

## 2 Materials and methods

### 2.1 Data sources and literature search strategy

For this scoping review, we followed the Preferred Reporting Items for Systematic Reviews and Meta-Analyses extension for Scoping Reviews Checklist (37). We performed a systematic search of the role of the complement system in TME on two main databases: PubMed and Web of Science. For the descriptors, we selected MeSH Terms in PubMed (“neoplasms”[MeSH Terms]) AND (“complement system proteins”[MeSH Terms]) and topics (Ts) in Web of Science (cancer AND (Complement system)) AND (OPEN ACCESS) AND (ARTICLE). We retrieved a total of 550 articles from PubMed and 69 from the Web of Science, and excluded duplicate articles using Excel (n=32).

### 2.2 Study eligibility – inclusion and exclusion criteria, and limitations

**Figure 2:**
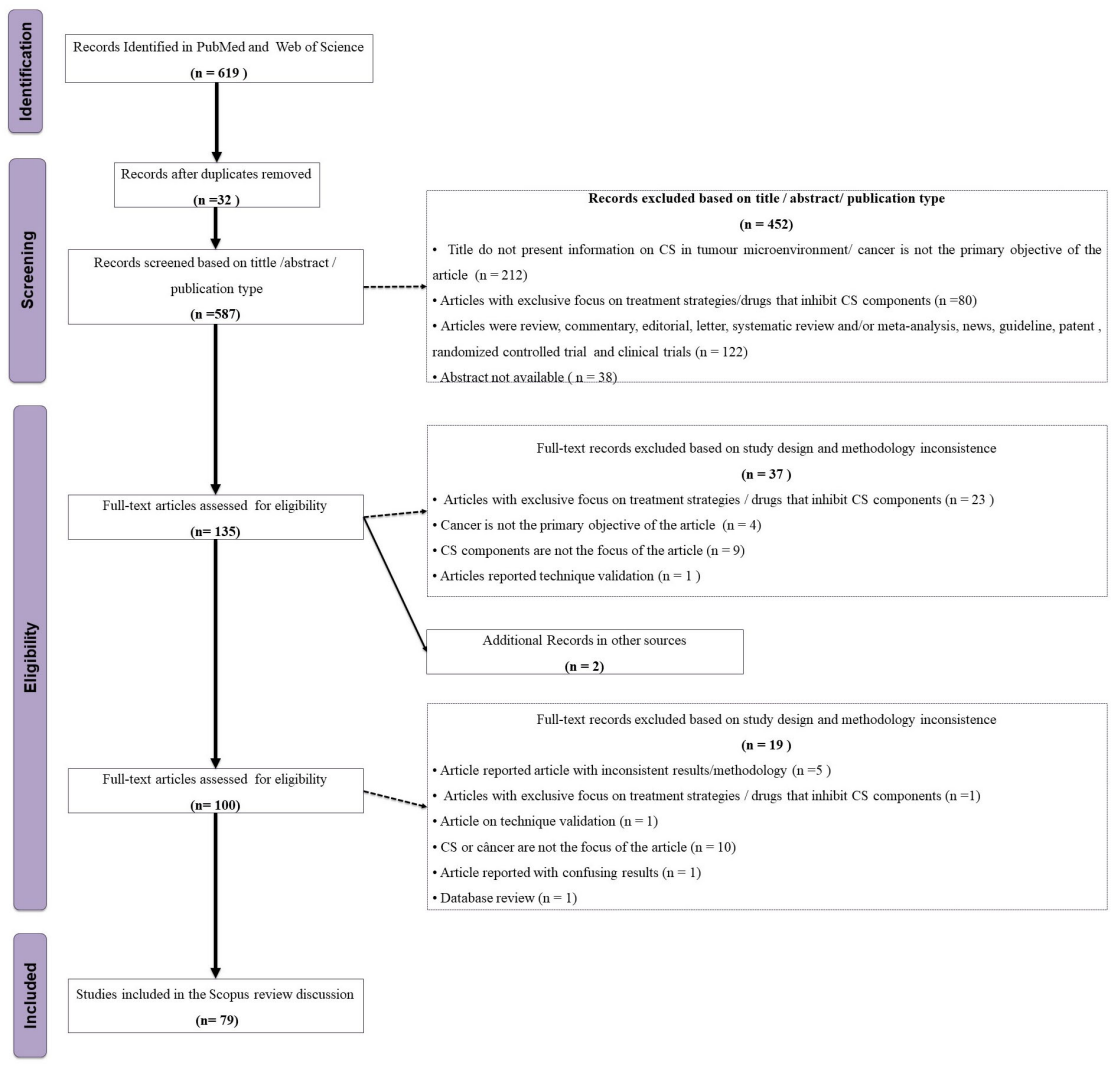
Flowchart of the study eligibility based on the PRISMA-ScR.

Independently of each other, two of us (CFO-T and AGM) screened the titles and abstracts of 619 articles for the following inclusion criteria: articles in English, with available abstract, open access, in humans or animal models, *in vivo*, *in vitro*, or *in silico*, and published from November 26th, 2010 to November 26th, 2020 (**Figure 2**). Exclusion criteria were: articles whose subjects were not components of the CS and cancer; studies classified either as review, commentary, editorial, letter, systematic review and/or meta-analysis, news, guidelines, clinical trials/controlled trials, and patents. We further excluded articles without an abstract and those with an exclusive focus on treatment strategies/drugs that inhibit CS components, retrieving a total of 135 articles, grouped according to the investigated topic. Next, five of us (CFO-T, HMBSP, NMDLS, PDF, and TPB) distributed the 135 articles among themselves according to their area of expertise and assessed their full content. We discussed and resolved any doubts by consensus, retrieving 45 studies for the final analysis (**Figure 2**, **Supplementary Table 1**). We extracted the following information from the selected studies: first author’s name, year of publication, title, experimental model, cancer type, complement components, anti/pro-tumoral role of complement components in the cancer context, and experimental methods. This review is limited by the inclusion of articles published up to 2020 and accessible through open access. As a result, relevant studies published after this period or behind paywalls may not have been considered. Additionally, as with any scoping review that relies on keyword searches in databases, some articles may have been unintentionally excluded due to variations in search terms or database indexing. Nevertheless, we made every effort to incorporate all relevant studies within the defined scope of this review.

## 3 Results

### 3.1 C1q complex

The C1 complex is composed of three proteins, C1q, C1r, and C1s. C1q is the recognition unit which is bound to two molecules each of the serine proteases C1r and C1s (C1q-C1r2-C1s2) (38, 39). C1q has a structural protein configuration consisting of a globular region and collagen-like domain. The globular region comprises three different subunits, A, B, and C, which form a trimer that is capable of recognizing a wide range of ligands. Apart from its role in CP activation, C1q interacts with complement receptor 1 (CR1), promoting phagocytosis of opsonized elements by mononuclear phagocytes and regulating their cytokine profile. C1q-stimulated macrophages upregulate pro-phagocytic genes, enhancing their phagocytic activity and altering cytokine expression, thereby suppressing the expression of IL-1 (interleukins 1 alpha and beta) and increasing secretion of interleukin-10 (IL-10), IL-1 receptor antagonist, monocyte chemoattractant protein-1, and interleukin-6 (IL-6) (40–42). It also plays an important anti-inflammatory role in maintaining homeostasis by aiding immune cells such as monocytes/macrophages and red blood cells in clearing immune complexes and apoptotic cells. Below we present our principal results, regarding C1q’s CP-independent role in cancer.

#### 3.1.1 C1q and C1r/C1s gene expression and protein levels in cancer

**Figure 3:**
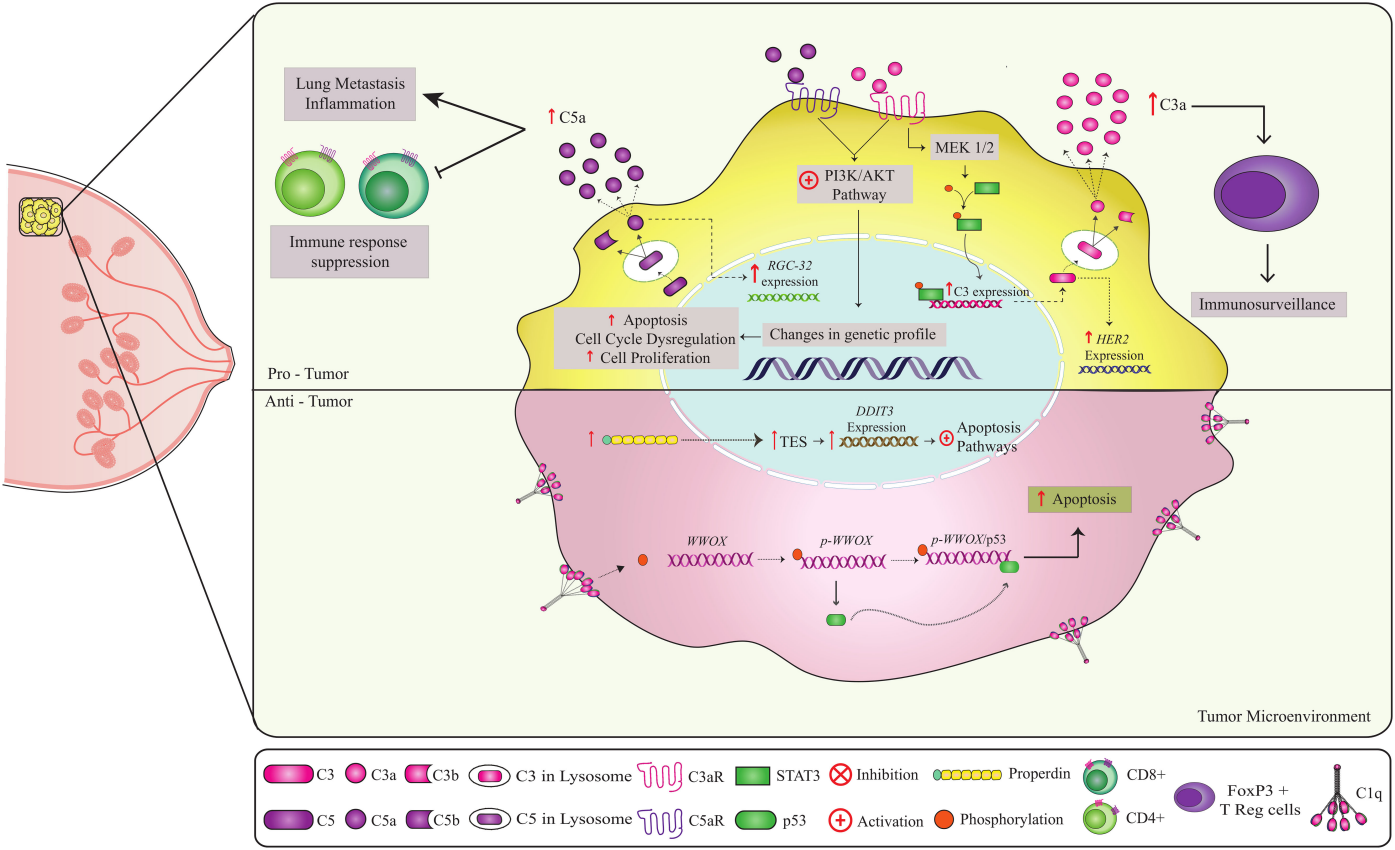
Non-canonical pathways involving complement components in breast cancer. Complement components play a role in both anti-tumor and pro-tumor responses. C1q and properdin have anti-tumor effects in breast cancer (51, 53, 91, 92). Binding of C1q to the cell at the breast tumor membrane leads to Tyr33 site phosphorylation of the WWOX protein, enabling the binding of p53 to *WWOX gene*. The p-WWOx-p53 complex is carried to the mitochondria and nucleus, where it activates apoptotic pathways (51). The high expression of intracellular properdin in tumor cells induces stress on the endoplasmic reticulum, leading to the expression of molecules that act in the increase of TES transcription, causing an increase in the expression of the DDIT3 gene that activates apoptotic pathways. Thus, both C1q and properdin contribute to the elimination of tumor cells. In contrast, on the C3a-C3aR and C5a-C5aR axes, CD46 and CD59 may have a pro-tumor role. Activation of the C3a-C3aR axis leads to PI3K/AKT and MEK1/2 pathway activation (63, 64). The PI3K/AKT pathway is a key regulator of the cellular processes involved in cell growth, metabolism, motility, survival, and apoptosis. Uncontrolled activation of this pathway promotes tumor cell survival and proliferation, leading to tumor progression and resistance to cancer therapy. Intracellular production of C5a leads to the C5a-C5aR axis, which can activate the PI3K/AKT pathway. C5a also promotes overexpression of the *RGC-32* gene (complement response gene 32), which controls cell cycle progression. High expression of the regulators CD46 and CD55 can potentially worsen prognosis and inhibit apoptosis, respectively. In addition, C3 has been shown to be involved in tumor immunosurveillance, probably through interactions with FOXP3+ T (Tregs), which are essential for the maintenance of immune tolerance and immune homeostasis. Thus, the combined action of these components can have a crucial effect on clinical outcomes.

C1q-deficient mice lacking the *C1qa* gene exhibited reduced B16 melanoma tumor mass and vascular density, as well as prolonged survival. C1q further induces proliferation, migration and murine melanoma cell adhesion, corroborating the hypothesis that C1q promotes melanoma progression *in vivo* independent of the CP activation (43). In line with this, tumor-associated macrophages (TAMs), resembling M2-like macrophages, as well as monocytoid B cells, contribute to elevated C1q levels in the TME. C1q-producing TAMs were associated with an immunosuppressed TME in clear cell renal cell carcinoma (ccRCC), characterized by high expression of immune-checkpoint molecules (PD1, LAG3, PDL1 and PDL2) (44). High C1q concentrations also occur in the TME of both human low-grade gliomas (LGGs) and glioblastoma multiforme (GBMs). In GBM, C1q was specifically associated with vascular endothelial cells, and its source could again be attributed to TAMs (45). C1q protein was diffusely present in the TME and expressed by TAMs in basal-like breast cancer and lung adenocarcinoma, whereas in clear cell renal cell carcinoma it was found in the small vessels and on the cell membrane of tumor cells - besides the TME. Expression of all three genes encoding C1q chains was associated with tumor growth and a poor prognosis in these types of lung and kidney cancer, despite representing a favorable prognosis in Her-2 positive and basal-type breast cancer (**Figure 3**) (46). Notably, there was no evidence of C3d nor C4d immunoreactivity and thus for complement activation in gliomas (45). In fact, deposition of other CS components seems to be infrequent in most tumors, suggesting that C1q operates in the TME independently of CP activation (43).

#### 3.1.2 C1q or C1r/C1s NCP signaling in cancer

*In vitro* studies have shown that C1q binds to high molecular weight hyaluronan (HMW-HA) in the extracellular matrix, triggering adhesion and proliferation of mesothelioma cells. This occurs through an increased phosphorylation of ERK1/ERK2 (extracellular signal-regulated kinases), SAPK/JNK, and p38, regardless of CP activation in a NCP manner (47). In a similar way, C1q has been shown to bind *in vitro* to discoidin domain tyrosine kinase receptors (DDRs) at the membrane of hepatocellular carcinoma cell lines. The C1q-DDR complex interacts with MMP2 and MMP9, two zinc-dependent matrix metalloproteinases that degrade extracellular matrix, promoting tumoral cell migration and tissue invasion (48). These data support the idea that C1q may increase the aggressiveness of liver cancer, as well as of mesothelioma, though through different mechanisms. Yet in breast and prostate cancer cell lines, extracellular C1q was involved in initiating a signaling pathway that activates the expression of the *WWOX* (WW domain-containing oxidoreductase) tumor-suppressor gene. In these particular tumor types, diminished C1q deposition was associated with an enhanced cell proliferation and less apoptosis (**Figure 3**) (22, 23).

In contrast to previous examples, cutaneous squamous cell (cSCC) lines exhibit high expression levels of *C1r* and *C1s* mRNA and protein, while lacking expression of genes producing C1q proteins. These cell lines secrete C1s and C1r, which leads to their activation in a C1q-independent manner. Consequently, activation of latent MMP9 occurs, accompanied by inhibition of extracellular signal-related kinase 1/2 and Akt activation (49). These findings suggest a potential role for these serine proteases in promoting cSCC tumor growth, angiogenesis, and metastasis.

### 3.2 C3 component

The canonical AP can contribute to up to 80% of the overall complement response and remains active even after activation of the CP and LP. These pathways converge to form and activate C3 convertase, which is a central step of the cascade that leads to the cleavage of C3, producing C3a and C3b. Moreover, AP C3 convertase amplifies C3b deposition on cell membranes, facilitating immune surveillance. C3a acts as a potent anaphylatoxin and induces chemotaxis, cell activation, and inflammation by binding to the C3aR receptor. The conformational change from C3 to C3b exposes previously hidden binding sites, enabling distinct interactions and efficient immune surveillance. Interactions between C3b and complement receptors/regulators, such as CR1/CD35 and CD46, was shown to lead to its degradation into iC3b and/or C3dg fragments, allowing immune adhesion, phagocytosis, and adaptive stimulation (**Figure 1**) (6).

#### 3.2.1 C3 gene expression and protein levels in cancer

In the neuT mouse model of breast cancer, C3 accumulates specifically in the vessels and stroma undergoing tumorigenesis in the mammary gland, while remaining absent in the surrounding tissue (**Figure 3**). These cancer cells express high levels of *Her2* and of the complement regulator CD55, preventing C3 cleavage by the C3 convertase. In the neuT C3-/- knockout mice, autochthonous mammary carcinomas started earlier and grew faster, reaching larger volumes in less time and presenting greater multiplicity, sooner seeding lung metastases. Tumors in these C3−/− mice showed 6- to 8-fold higher levels of Her2/neu expression. Thus, C3 may limit Her2 expression and, consequently, cell proliferation. The número of regulatory T cells (Tregs) did also increase in tumors from neuT-C3−/− mice, contributing to a more immunosuppressive environment and favoring tumor growth. Although the number of tumor vessels was similar between neuT and neuT-C3−/− mice, the vessels in C3-deficient tumors were wider and more permeable, resulting in better nutrient and oxygen supply to the tumor (**Figure 3**) (50).

#### 3.2.2 C3 and C3aR NCP signaling in cancer

**Figure 4:**
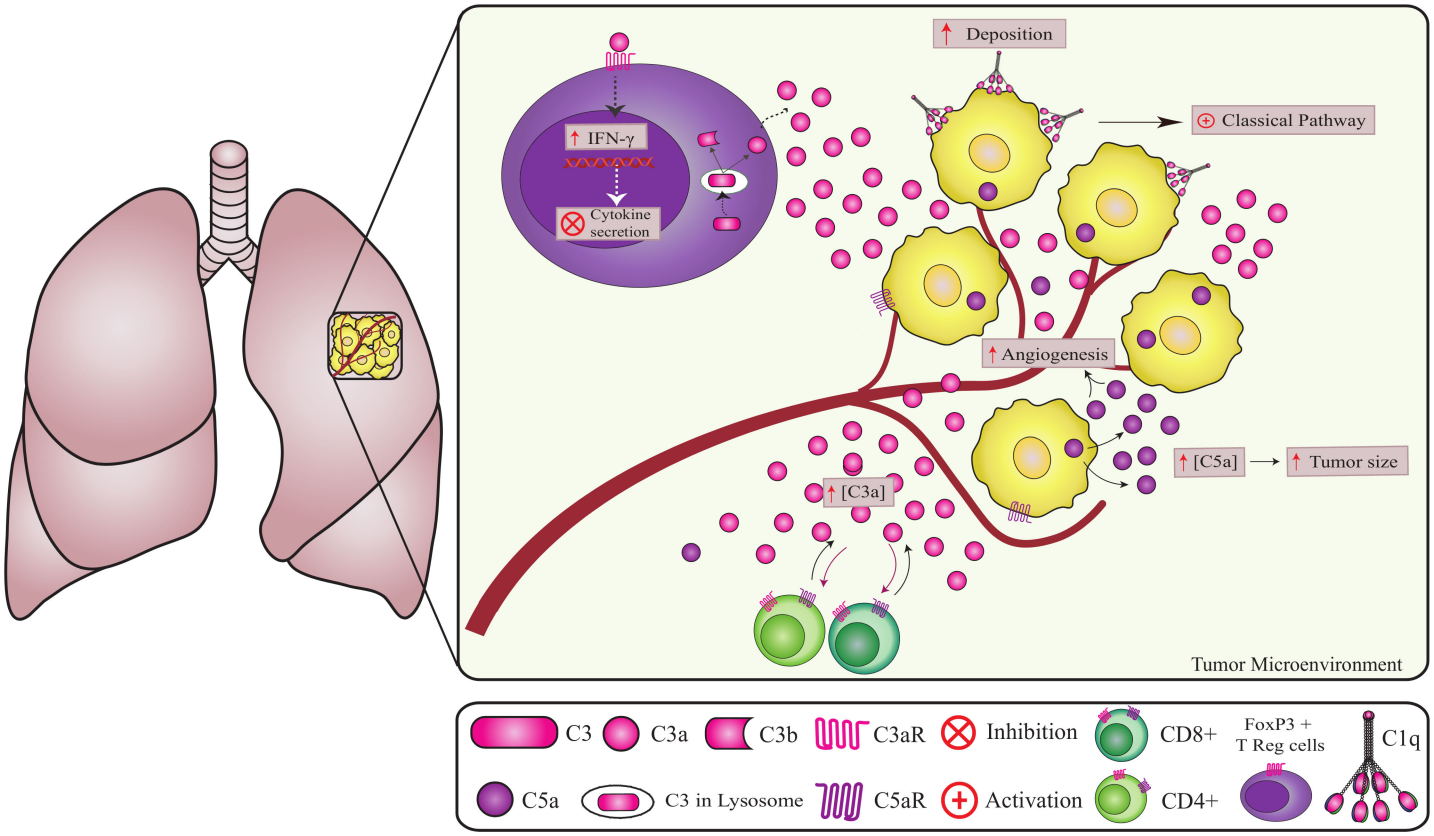
Non-canonical pathways involving complement components in lung cancer. Complement components play a crucial role in lung cancer progression. C1q promotes angiogenesis independent of CS activation (2). However, the origin of intracellular C5a in cancer cells remains unknown. Increased extracellular C5a levels lead to tumor growth and angiogenesis (72, 74). The high deposition of factor H on the tumor cell membrane contributes to tumor progression and can be considered a marker for lung adenocarcinomas. A high concentration of C3 in the TME reduces CD4 T cells, promoting metastasis and tumor growth. In addition, CD4+ T cells can produce and cleave C3 intracellularly via cathepsin leading to the regulation of IFNγ (interferon-gamma) secretion (60, 61).

In lung cancer, CD4+ T cells present intracellular C3 cleavage by cathepsin and C3a signaling inhibits the production of multiple cytokines by CD4+ T cells, independently of FOXP3+ Tregs (51) (**Figure 4**). *In vitro* studies revealed that iC3b is inactivated by prostate-specific antigen (PSA) in prostatic fluid, generating a novel 37 kDa fragment independently of factors H and I, smaller than that produced by the canonical pathways. Notably, the new iC3b fragments appear to promote an immunosuppressive prostatic tumor profile, although their biological function is still unclear (52).

C3a has the potential to activate ERK/MEK1-2 kinases promoting cell proliferation and apoptosis resistance, regulating synaptic plasticity and p53 phosphorylation, e.g. human bone osteosarcoma epithelial cells (U2-OS) exposed to normal serum exhibited increased ERK phosphorylation compared to U2-OS treated with heat-inactivated serum (53). In gastric cancer, deposition of C3 fragments on tumor cells activates the JAK2/STAT3 pathway, increasing levels of phosphorylated STAT3 (promoting cell proliferation and migration) and IL-6 (54). Ovarian cancer cells secrete high amounts of C3, whose non-canonical cleavage (independent of CP, AP and LP) leads to C3a production in the TME. C3a binding to C3aRs on the tumoral cell membrane activate the PI3K/AKT/mTOR pathway, boosting protein synthesis, cell proliferation, survival, and motility by increasing AKT mRNA levels and phosphorylating p85, a PI3K regulatory subunit (55). Similarly, in ovarian cancer mice models (C57BL/6), the C3a-C3aR interaction triggers PI3K/AKT pathway activation, enhancing C3 secretion, tumor growth, and proliferation via a C3-dependent autocrine loop (55). Finally, C3a-C3aR signaling through PI3K-AKT promote lung metastases of breast cancer in a murine model, modulating carcinoma-associated fibroblasts to increase secretion of pro-metastatic cytokines and expression of extracellular matrix components (**Figure 3**) (56).

### 3.3 C5 component in cancer

The final phase of the CS cascade starts with the formation of C5 convertase, which catalyzes the cleavage of the C5 component into C5a and C5b (**Figure 1**). C5a plays a role as a potent anaphylatoxin and chemoattractant for neutrophils, monocytes, and macrophages (57). Additionally, intracellular C5a modulates cytokine expression in various cell types, playing a role in activating coagulation pathways and inducing the formation of neutrophil extracellular traps (NETs). The C5a receptor (C5aR), a member of the rhodopsin family of G protein-coupled receptors, is predominantly expressed by myeloid cells in the lung and liver. Activation of C5aR triggers diverse local responses depending on the cell type, with implications in cancer, particularly in the generation and modulation of anti-apoptotic responses, among other functions (**Figure 1**) (58).

#### 3.3.1 C5 and C5aR gene expression and protein levels in cancer

In lung (59), colon (60), ovarian (36), and bile duct cancers (60), C5a production does not appear to be associated with the activation of the CP or AP, a matter that still needs to be clarified (59, 60). *In vitro*, lung cancer cells produced C5a independently of the expression of factor B, properdin, C1q, C1r, C1s, C1, and C4 (**Figure 4**) (59). However, C5a production was reduced by inhibitors of trypsin-like serine proteases. MASP-1, a serine protease from the LP with trypsin-like activity, might play a role in producing tumoral C5a. However, further experiments using inhibitors of LP serine proteases are required to confirm this possibility (59).

#### 3.3.2 C5a and C5aR NCP signaling in cancer

Similarly to C3, C5 activation plays a pro-tumoral role in different types of cancer. Certain types of cancer cells, such as those found in lung, colon, ovarian, and bile duct cancers, secrete C5a into the TME, independently of CP and AP, initiating an autocrine loop that enhances cell proliferation and fosters metastasis. Mice with lung cancer, if treated with C5aR antagonist, slow the growth of the tumor and decrease angiogenesis (59, 60).

In lung cancer tissue and cell lines, C5a contributes to an immunosuppressive microenvironment by recruiting myeloid-derived suppressor cells and inducing the expression of several immunomodulators, including arginase 1 (ARG1), cytotoxic T-lymphocyte-associated protein 4 (CTLA-4), IL-6, IL-10, lymphocyte-activation gene 3 (*LAG3*), and programmed cell death 1 ligand 1 (PD-L1 or CD274) (59). Similarly, in ovarian cancer, elevated levels of C5a impact the levels of immunoregulators in the TME, such as arginase, inducible nitric oxide synthase (iNOS), vascular endothelial growth factor (VEGF), and tumor necrosis factor alpha (TNF-α), thereby reducing the infiltration of CD4+ and CD8+ T cells and promoting tumor growth (36). High C5a levels stimulate linfoma with decreased infiltration of CD4+ and CD8+ T cells, while low levels of C5a in the TME decrease tumor progression, thus, the local concentration of C5a, independent of complement activation, seem to be critical to determine its role in tumor progression (61).

In human breast cancer cell lines, C5a induces the overexpression of the *RGCC* gene (response gene to complement 32 protein, involved in cell cycle progression), by activating the Akt pathway (**Figure 3**) (62). Additionally, C5a-C5aR signaling facilitates lung metastasis, a common occurrence in breast cancer, by suppressing the responses of CD8+ and CD4+ T cells and promoting the generation of regulatory T cells (Tregs) *in situ* (**Figure 3**) (63). The mechanism of this C5aR-mediated T cell suppression in the metastatic target involves the recruitment of immature myeloid cells and increased production of transforming growth factor β (TGF-β) and IL-10, favoring the generation of Treg and Th2-oriented responses that render CD8+ T cells, dysfunctional (**Figure 3**) (63).

In gastric cancer, C5a-C5aR recognition regulates p21 expression through the PI3K/AKT axis. Moreover, increased C5aR expression by the MKN1 and MKN7 gastric cancer cells enhances their invasiveness and promotes liver metastasis, which is associated with a poor prognosis (64). In these cells, C5a-C5aR signaling promotes the conversion of RhoA-GDP (RhoA-guanosine diphosphate) to RhoA-GTP (RhoA-guanosine triphosphate) in the cytosol, leading to cytoskeletal rearrangement and increased invasive capacity (65). In fact, in renal cell carcinoma the activation of the C5a-C5aR axis was indicated as a prognostic marker, with C5a stimulating ERK and PI3K-dependent invasion in renal cell carcinoma cells expressing C5aR (66, 67).

In metastatic pancreatic invasive ductal adenocarcinoma, podocalyxin-like protein 1 (PODXL1) activates C5aR on the cell membrane, increasing cellular motility. This activation confers invasive and metastatic properties to PDAC cells (68). Furthermore, C5aR1 signaling induces the secretion of chemokine C-C motif ligand 2 (CCL2) by melanoma cells. This attracts immunosuppressive populations of myeloid cells, supporting tumoral growth and facilitating the infiltration of leukocytes with an immunosuppressive profile in the TME. Conversely, C5aR2 plays a more limited yet beneficial role in restraining tumor growth (69).

In summary, C5a/C5aR signaling plays a significant pro-tumoral activity in several tumor types. Binding of C5a to C5aR in tumor cells correlates with reduced overall survival and recurrence-free survival, while also elevating the incidence of microvascular invasion and metastasis of gastric cancer and renal cell carcinoma (**Figures 1**, **3**, **4**) (66, 67).

### 3.4 The final complement lytic pathway in cancer

The final complement lytic pathway serves as the common intersection for the three canonical complement pathways, which culminate with the disruption of plasma membranes of target cells or microorganisms (**Figure 1**) (70). Complement regulators restrict CS activation on most tumors. However, sublytic MAC deposition on cancer cells raises intracellular Ca2+ levels, altering cellular functionality and causing cellular damage (70). MAC activity is crucial for maintaining cellular and tissue homeostasis and promoting protection against infections. It may also maintain tumor-associated inflammatory signaling, assuming a dual role in cancer (71, 72).

#### 3.4.1 The role of MAC and C7 in cancer

Exposure of cancer cells to sublytic levels of MAC alters the expression of G protein and Ca2+ signal transduction (ITPRIP, RGS16), transcription factors (EGR1, EGR2), inflammatory response genes (Interferon Regulatory Factor 1 - IRF1), as well as four other extracellular protein genes (Amphiregulin - AREG, C-X-C Motif Chemokine Ligand 1 - CXCL1, Matrix Metalloproteinase 3 - MMP3, and MMP13), impacting cell proliferation and survival (73). In this way, sublytic MAC deposition on cancer cells emerges as a potent stimulator of tumor progression.

C7 is an important protein in the MAC formation. Increased amounts of C7 protein in the nuclei of hepatocellular carcinoma cell lines promotes *in vivo* cell growth, by upregulating late SV40 factor (LSF-1) protein levels and the expression of stemness factors such as POU Class 5 Homeobox 1, SRY-Box Transcription Factor 2, and MYC Proto-Oncogene Transcription Factor (encoded by *OCT4, SOX2*, and *MYC*, respectively) (74). These alterations in gene expression sustain the replication capacity of liver tumor-initiating cells, while suppression of C7 inhibited the establishment of human tumors in NOD/SCID (non obese diabetic/severe combined immunodeficiency) mice (74).

On the other hand, in non-small cell lung cancer (NSCLC), C7 overexpression suppresses colony formation *in vitro* and lower C7 expression was associated with worse outcome, advanced clinical stage and grade, increased likelihood of relapse and death (75). Therefore, the anti- or pro-tumorigenic activity of C7 is influenced by the different cancer types. While these components act as independent drivers of tumor immune response and surveillance, further investigation is needed to clarify their *in vivo* effects on carcinogenesis and tumor progression.

### 3.5 Complement regulators in cancer

Complement activation leads to various biological processes extending beyond its protective function and can, in some cases, pose harm. Therefore, safeguarding self-tissues from complement-mediated damage is crucial, achieved through the actions of several soluble regulators or membrane-bound counterparts. Different cell types rely on a combination of complement regulatory proteins (CRPs) to modulate the cascade at various points and in diverse manners. Noteworthy membrane-bound CRPs in tumors include CD46, CD55, and CD59, while soluble ones encompass factor I, factor H, and properdin (76, 77).Although CD46, CD55, and CD59 are pivotal complement regulators expressed across most cell types and tumor cells, they exhibit a double-edged sword role. While they avert complement-mediated autologous lysis in normal cells, their aberrant overexpression impedes complement-mediated lysis, thereby fostering tumor cell survival and progression. Consequently, membrane-bound CRPs may serve as biomarkers of malignant transformation (78–81).

CD59, a prevalent regulator expressed in most tumor cells, is a glycosylphosphatidylinositol (GPI)-anchored membrane protein that impedes C9 polymerization and the attachment of C9 units to the C5b-8 complex and MAC through its physical incorporation into the complex (79), thereby preventing membrane disruption and cell lysis. CD55, referred to as a complement decay-accelerating factor (DAF), expedites the decay of C3 and C5 convertases by swiftly dissociating the catalytic subunit C2a or Bb from the cell surface, thus preventing the generation of anaphylatoxins, opsonins, and MAC. Additionally, CD55 can recognize C4b and C3b fragments produced during C4 or C3 activation (78). These CRPs exert significant modulation over complement activity and may profoundly influence tumor progression.

The factor H (FH) is a plasma glycoprotein that acts as a soluble inhibitor of complement, where its binding to self markers such as glycan structures prevents complement activation and amplification on cell surfaces (82, 83). FH ensures the complement system spares host tissues from damage, by destabilizing the AP C3 convertase complex (C3bBb). Its dysfunction in age-related macular degeneration and atypical hemolytic uremic syndrome highlights its importance in maintaining the balance between effective immunity and self-damage. In contrast, properdin stabilizes C3bBb, amplifying pathogen opsonization, inflammation, and MAC formation. Properdin is unique as one of the few complement components that act as a positive regulator in the system (84). As a plasma glycoprotein, the complement factor properdin (CFP) is the only known positive regulator of the CS. It plays a crucial role in stabilizing surface-bound alternative pathway (AP) C3 and C5 convertases (64, 85).

#### 3.5.1 CD59, CD55 and CD46 gene expression and protein levels in cancer

Head and neck cancer cells exhibit high expression of CD46, CD55, and CD59 (86). CD59 is highly expressed on tumoral cells, protecting them against complement lysis, and aiding in immune evasion. CD59 expression correlated with TAM infiltration in pancreatic cancer tissues. Pancreatic cancer-educated macrophages upregulate CD59 expression on pancreatic cancer cell lines through STAT3 phosphorylation via the IL-6R/STAT3 signaling pathway and protect them from complement-dependent cytotoxicity (81). Similarly, in human ovarian A2780 cancer cells, IL-6 stimulates CD59 expression at low concentrations, but at high concentrations or with IL-8, it presents a post-transcriptional inhibitory effect (87). Indeed, CD59 immunoreactivity is detected in up to 50% of ovarian tumors and at the border areas between normal and malignant tissue (87). Similarly, increased CD55 and CD59 expression was observed on cell membranes and in the TME of ovarian and uterine cancers (80, 88). Additionally, CD55 overexpression predicts poorer clinical outcomes in colorectal cancer, with stromal CD55 overexpression correlating with unfavorable prognostic markers (78). Beyond its role in inhibiting canonical complement action within the TME, CD59 was shown to impede apoptosis of breast cancer cells, thereby contributing to tumor development (79).

High CD46 expression is an unfavorable prognostic factor associated with lower relapse-free survival in breast cancer (89). Notably, the *CD46* gene promoter has two binding sites for STAT3. If induced by STAT3 and IL-6, CD46 expression protects cancer cells against complement lysis and fosters a pro-tumoral profile in breast and prostate cancers (89). On the other hand, low CD46 expression rates in cervical cancer correlate with increased patient´s survival (90). CD46 shifts its expression profile in cervical cancer cells, compared to normal female reproductive tissue, suggesting its involvement in tumorigenesis.

#### 3.5.2 Complosomes with FH and properdin in cancer

As is the case with complosome involving C7, intranuclear FH promotes via LSF-1, the expression of *NANOG*, *OCT4*, *SOX2*, and *MYC* genes. On their turn, these stemness factors promote liver tumor formation and growth *in vivo* (**Figure 1**). Notably, increased FH expression, particularly within the nucleus, suggests enrichment in tumor cells (74). Similarly, FH and its isoform FHL-1 expression is specifically induced during cutaneous carcinogenesis (cSCC) and exert effects on intracellular pathways, being negatively regulated by the inhibition of ERK1/2, p38, and MAPK pathways, while being upregulated by IFNγ, IL-1b, TGF-α, TGF-β, and TNF-α. FH/FHL-1 silencing inhibited tumoral cell proliferation and migration, pointing to a critical protumorigenic role (91).

In the complosome network, properdin presented an anti-tumoral role through a novel tumor suppressor pathway identified in breast cancer (**Figures 1**, **3**). High expression of intracellular properdin induces the transcription of the Testin LIM domain protein (TES), subsequently leading to increased expression of DDIT3 (DNA-Damage-Inducible Transcript 3), a pro-apoptotic transcription factor associated with the endoplasmic reticulum stress response. Consequently, increased properdin expression modulates cell apoptosis, playing an anti-tumoral role in breast cancer (**Figure 3**) (92). Furthermore, *in vitro* studies have demonstrated that bone marrow-derived macrophages (BMDM) from properdin-deficient mice can stimulate B16F10 melanoma cell lines. This properdin deficiency results in low levels of IL-1b mRNA and higher levels of arginase-1, monocyte chemotactic protein-1 (MCP-1), and IL-10 mRNAs, suggesting that a properdin-deficient tumor microenvironment may induce a profile of M2 macrophages with pro-tumoral activity (92).

Although there is currently no significant association between properdin expression and cancer prognosis, this regulator plays an important anti-tumoral role in complosome pathways in breast cancer³ (93). In summary, FH acts as a pro-tumoral factor in hepatocellular carcinoma, while properdin serves as an anti-tumoral factor suppressing breast cancer/melanoma. These two CRPs likely play a role in complosome pathways by modulating tumor formation and growth.

## 4 Discussion

Research over the past decade has firmly established that CS components play a pivotal role in driving hallmark features of cancer. These elements contribute to critical tumor processes such as angiogenesis, invasion, metastasis, immunomodulation, metabolic reprogramming, microenvironmental remodeling, unchecked proliferation, and therapy resistance. While canonical complement activation pathways are implicated in many of these mechanisms, emerging evidence highlights the substantial involvement of CS components through activation-independent pathways. Its role in cancer is context-dependent, shaped by its cellular source, interaction partners, and downstream signaling pathways.

We found little evidence for complosome activity in cancer, but rather for NCP-mediated activation of intracellular signaling pathways, suppressing tumors in certain settings (e.g., for C1q, by activating *WWOX* in breast and prostate cancer cells (22, 23), and more commonly driving malignancy by stromal interactions, receptor signaling, and matrix remodeling, (e.g. C1q by engaging receptors as DDR1 and HA to activate MAPK signaling or modulate MMPs, fostering tumor aggression) (47, 48). A recent study published after the timeframe of our scoping review described a pro-tumorigenic C1q-driven mechanism in pleural mesothelioma (PM). The researchers identified a functional interplay between hyaluronidase-2 (HYAL2)—a prognostic marker associated with poor PM outcomes—and the hyaluronic acid-binding protein gC1qR (globular C1q receptor/HABP1/p32). C1q binding to hyaluronic acid (HA) via gC1qR activates intracellular signaling pathways that up-regulate the expression of both *HYAL2* and hyaluronan synthase *HAS3* mRNA and protein expression. Through this NCP, C1q both increases HA synthesis and stimulates its degradation into low-molecular-weight fragments, creating a feedback loop that supports tumor growth and maintenance (94, 95).

Beside complement regulators and receptors, components normally produced in the liver, as C1q, C1r, C1s, C3 and C5 are locally synthesized and deposited in different tumor types and in the stroma regardless of complement activation, being predominantly tumorigenic in the majority of cancer types. C3a-C3aR and C5a-C5aR binding activate the PI3K/AKT/mTOR pathways, which promote cell proliferation, protein synthesis, survival and motility. Concurrently, C3 cleavage fragments stimulate the JAK2/STAT3 and ERK pathways, driving cellular reprogramming that promotes tumor progression. These pathways collectively orchestrate pro-tumorigenic changes such as enhanced survival, proliferation, and immune evasion (56).

Thus, tumor fate depends on all the dynamic interactions between complement components, immune cells, and signaling pathways in the TME. A tumor-friendly TME would probably present one or more of the following features: PI3K/AKT and MEK1/2 signaling via *C3a-C3aR* and/or *C5a-C5aR* axes, JAK2/STAT3 activation driven by tumor-deposited C3 fragments, promoting growth/metastasis, anti-apoptotic effects via CD59-mediated inhibition of terminal complement complexes, *C5a*-induced Akt-dependent oncogenic RGC-32 expression, overexpression of CD55, CD46, and CD59 on tumor cells, Factor H overexpression, enhancing stemness (via *LSF-1*) and angiogenesis. On the contrary, TME-driven tumor suppression would include *C1q*-mediated phosphorylation of the WWOX tumor suppressor, *Properdin*-dependent activation of the TES-DDIT3 tumor suppressor pathway, CD4+/CD8+ T-cell infiltration due to high intratumoral C3 expression and C3-mediated inhibition of *HER2* oncogene expression.

While no studies meeting our inclusion criteria (Section 2.2) directly demonstrated LP components influencing carcinogenesis via complosomes or NCPs, two references are worth mentioning. Ficolin-2 was found to inhibit epithelial–mesenchymal transition in HCC, both *in vivo* and *in vitro*, by decreasing TGF beta levels and phosphorylation of Smad2 and Smad3 (96). FCN-2 was also shown to interact with Toll-like receptor 4 (TLR4) and drive macrophage polarization toward the M1 phenotype. This activation enhanced antigen presentation to CD8+ T lymphocytes, ultimately reducing tumor growth (CT26 colon carcinoma, Lewis lung carcinoma, and Hca-f hepatocarcinoma) across three distinct murine models (BALB/c, C57BL/6, and C3H/He). Notably, FCN-A knockout mice exhibited accelerated CT26 colon and Lewis lung carcinoma progression – a phenomenon reversed by FCN-2 or FCN-A supplementation (97). In fact, LP components seem very relevant in oncogenic processes (98, 99). Their absence from currently characterized carcinogenic pathways reflects the paucity of mechanistic studies, underscoring the need for systematic functional characterization of these molecules in oncogenic processes.

A central unresolved question persists: do CS components drive tumorigenesis, or are they merely bystanders in the TME? While some studies suggest individual CS elements may foster tumor-friendly niches, this perspective conflicts with cancer’s multifactorial etiology. Instead, we propose that CS components act as dynamic regulators of the TME, through direct involvement in complement activation cascades, in NCP and complosomes leading to receptor signaling, and crosstalk with stromal cells. Critically, the TME itself dictates whether CS components promote or suppress tumor progression. For example, tumor cells and TME-associated immune cells (e.g., macrophages, monocytes) can locally produce CS proteins. These elements may then paradoxically support or antagonize tumor growth depending on contextual factors like tumor type, stage, and immune landscape (44, 45). This complexity underscores the need for cancer-specific investigations to unravel the precise role and therapeutic potential of each complement component.
