# Supplementary material for: Non-canonical extracellular complement pathways and the complosome paradigm in cancer: a scoping review
Source: Front Immunol. 2025 Apr 30;16:1519465. doi: 10.3389/fimmu.2025.1519465 (PMC12075386; doi:10.3389/fimmu.2025.1519465)
Supplement: Supplementary file 1 [file Table1.docx]

**Supplementary Table 1.** Articles included in the scoping review showing anti- or pro-tumor roles of complement system in non-canonical pathways

| Complement component | Type of cancer | Study design | Effect in the cancer | Unusual complement pathway | Reference |
| --- | --- | --- | --- | --- | --- |
| Pro-tumor Role | | | | | |
| C1q complex |  |  |  |  |  |
|  | Melanoma | Human tissue  Mice models  Cell line | C1q deposition without local complement activation | n.a | [43] |
|  | Lung adenocarcinoma | Human tissue  Mice models  Cell line | C1q deposition without local complement activation | n.a | [43] |
|  | Breast | Human tissue  Mice models  Cell line | C1q deposition without local complement activation | n.a | [43] |
|  | Pancreatic adenocarcinoma | Human tissue  Mice models  Cell line | C1q deposition without local complement activation | n.a | [43] |
|  | Cervical adenocarcinoma | Human tissue  Mouse model  Cell line | C1q deposition without local complement activation | n.a | [43] |
|  | Glioblastomas multiforme | *In silico* Bioinformatics  Human Tissue | C1q associated with vascular endothelium, tumor development without complement activation | n.a | [45] |
|  | Low-grade gliomas | *In silico* Bioinformatics  Human Tissue | C1q associated with vascular endothelium, tumor development without complement activation | n.a | [45] |
|  | Clear cell renal cell carcinoma | *In silico* Bioinformatics  Human Tissue  Cell line  Mouse model | C1q-producing TAMs exhibited an immunosuppressed microenvironment  Classical complement pathway is activated *in situ* | C1q production by TAMs | [46] |
|  | Malignant pleural mesothelioma | Human Tissue | C1q + Hyaluronic Acid:  Induced adhesion and proliferation of MES via enhancement of ERK1/2, SAPK/JNK, and p38 | C1q-ERK1/2  SAPK/JNK  p38 | [47] |
|  | Hepatocellular carcinoma | Cell line | C1q induces direct activation and upregulation of DDR1, with enhanced migration and invasion of HepG2 cells | C1q-DDR1 | [48] |
|  | Cutaneous squamous cell carcinoma (cSCC) | Cell line | Cleavage and activation of C1s in the absence of C1q, activates latent MMP-9 | C1s-MMP-9 | [49] |
| C3 and C3a |  |  |  |  |  |
|  | Osteosarcoma | Cell line | C3 activation leads to ERK phosphorylation and this is associated with complement-mediated (AP) production of VEGF-A and FGF1, angiogenic growth factors | ERK phosphorylation | [53] |
|  | Prostate cancer | Human | High-level of C3 in the prostatic fluid  Purified PSA was able to cleave iC3b into a new fragment | PSA cleaves iC3b | [52] |
|  | Ovarian cancer | Mouse models | C3a activates its receptor on cancer cells via PI3K/AKT | PI3K/AKT | [55] |
|  | Breast cancer | Mouse model | C3 activation at the tumor site may positively regulate *Her^2^*  Her2^+^ C3 cells was involved in tumor immunosurveillance probably due to interactions with FoxP3^+^ T (T_regs_) | C3-*Her^2^*  FoxP3^+^ T (T_regs_) | [50] |
|  | Breast cancer | Mouse models | C3a increases the secretion of pro-metastatic cytokines and expression of extracellular matrix components through the activation of PI3K-AKT signaling pathway  C3a-C3aR axis promotes lung metastases of breast cancer through modulation of carcinoma-associated fibroblasts | C3a-PI3K/AKT | [55] |
|  | Lung Cancer | Mouse models | C3 signaling has the ability to inhibit directly or indirectly the production of multiple cytokines by CD4^+^ T cells independent of FoxP3^+^ T_regs_ | C3-CD4^+^ T cells  FoxP3^+^ T_regs_ | [51] |
|  | Gastric cancer | Human | C3 acts as an upstream regulator of JAK2/STAT3 activation. Localized activation and deposition of C3 may play a role in tumor growth and metastasis process | C3-JAK2/STAT3 pathway | [56] |
| C5a and C5aR |  |  |  |  |  |
|  | Bile duct cancer | Cell line | Release of C5a by cancer cells represents a new self-activation circuit via C5a-C5aR in cancer cells that express C5aR, independently of complement activation  C5a induces recruitment of MDSCs and angiogenesis,  favoring cancer progression | Self-activation circuit of C5a by via pC5a-C5aR | [60] |
|  | Gastric Cancer | Human tissue  Cell line  Mouse model | C5a promoted the expression of p-PI3K/p-AKT  Suppression of CD4^+^ CD8^+^ T cells | pPI3K/p-AKT | [64] |
|  | Breast Cancer | Mouse model  Cell line | C5aR induces metastasis by suppressing CD8^+^ and CD4^+^ T cell responses in the lungs | Suppressed CD4^+^ and CD8^+^ T cells response | [63] |
|  | Ovary cancer | Cell line  Mouse model | High C5a concentrations were related to tumor growth and decreased CD8^+^ and CD4^+^ T cells infiltrates the interaction  The interaction C5a-VEGF165 increases tumor growth | Decreased CD4^+^ and CD8^+^ infiltration | [36] |
|  | Pancreatic invasive ductal adenocarcinoma | Human Cell line  Human tissue  Mouse model | The interaction between C5aR and PODXL1 leads to invasion and tumor metastasis with poor prognosis | C5aR-PODXL1 | [68] |
|  | Melanoma | Cell line | C5aR1 signaling promote melanoma growth by the infiltration of immunosuppressive leukocyte populations into the tumor microenvironment | n.a | [69] |
|  | Breast Cancer | Cell line | Blockade of C5a receptor markedly reduced the expression of *RGC-32* and the proliferation of breast cancer cells  Silencing of *RGC-32* expression reduced the proliferation of breast cancer cells induced by C5a | *RGC-32* gene | [62] |
|  | Clear cell renal cell carcinoma | Cell line  Human tissue | C5a-C5aR axis related to worse prognostic, C5a triggers the ERK and PI3K dependent invasion of renal cell carcinoma expressing C5aR | ERK/PI3K | [67] |
|  | Lung cancer | Human serum | Exclusive production of C5a with no concomitant expression of other complement components | n.a | [59] |
|  | Gastric cancer | Cell line | C5aR expression facilitates liver metastasis  C5a-C5aR signal increases the conversion of RhoA-GDP to RhoA-GTP in the cytosol, inducing a cytoskeletal rearrangement and increase invasiveness capacity of the tumor | RhoA-GDP  RhoA-GTP | [65] |
|  | Renal cell carcinoma | Human tissue | The C5a-ERk-PI3K pathway triggers the process of invasion of renal carcinoma cells, without the activation of complement pathways | ERK/PI3K | [66] |
|  | Ovarian cancer | Mouse model | High levels of C5a stimulates tumor growth and decreases infiltration of CD4^+^ and CD8^+^ T cells | CD4^+^ and CD8^+^ suppressing | [36] |
| C5b-9  MAC |  |  |  |  |  |
|  | Colon carcinoma | Cell line | Sublytic MAC resulted in changes of cell gene expression associated to G protein and Ca2+ signal transduction (ITPRIP, RGS16), transcription factors (EGR1, EGR2) and inflammatory responses (IRF1)  Additionally it affects four other extracellular protein-related genes (AREG, CXCL1, MMP3, MMP13) | ITPRIP, RGS16,EGR1, EGR2, IRF1, AREG, CXCL1, MMP3, MMP13 | [73] |
| C7 |  |  |  |  |  |
|  | Liver cancer | Liver cancer cells | C7 overexpression promoted tumor cell growth, upregulated LSF protein levels leading to upregulation of stemness factors expression such as Oct4, Sox2 and c-Myc, sustaining stemness and replication capacity in liver tumor-initiating cells | Late SV40 Factor or *TFCP2* gene, Oct4, Sox2 and c-Myc | [74] |
| Factor H |  |  |  |  |  |
|  | Liver cancer | Human tissue  Mouse model | Positive regulation of stemness factors via LSF-1 | Late SV40 Factor or *TFCP2* gene | [74] |
|  | Cutaneous Squamous Cell | Human tissue  Human serum levels | FH and its FHL-1 isoform are down-regulated in the ERK1/2, p38 and MAPK pathways  Complement activation | ERK1/2  p38  MAPK | [91] |
| CD46 |  |  |  |  |  |
|  | Breast cancer | Cell line | *CD46 mRNA* expression is induced by interleukin-6  Activated STAT3 signaling induces the activation of the *CD46* promoter | STAT3 and IL-6 | [89] |
|  | Prostate cancer | Cell line | *CD46 mRNA* expression is induced by interleukin-6  Activated STAT3 signaling induces the activation of the *CD46* promoter | STAT3 and IL-6 | [89] |
|  | Head and neck squamous cell carcinoma | Cell line  Human tissue | Occur strong expression of the regulator in cell line of head and neck cancer as well as in newly derived tumor tissue, metastatic lymph node tissue, tumor infiltration and peripheral blood lymphocytes | n.a | [86] |
|  | Breast cancer | Cell line | Expression of CD46 in related to worse prognosis | n.a | [89] |
| CD55 |  |  |  |  |  |
|  | Head and neck cancer | Cell cultures  Human tissue | Strong expression of the CD59 in cancer as well as in the newly derived tumor tissue, metastatic lymph node tissue, tumor infiltration and peripheral blood lymphocytes | n.a | [86] |
|  | Ovarian carcinoma | Ovarian carcinoma Cell line | Increased expression of the CD55 protein | n.a | [87] |
|  | Ovary and corpus uteri tumors | Cell line  Human tissue | CD55 present predominantly on cancerous tubules | n.a | [88] |
|  | Colorectal | Cells lines | High CD55 expression associated with lower patient survival | n.a | [78] |
|  | Ovary and corpus uteri tumors | Human tissue | Increased expression of CD55 in uterine tumors and decreased expression in ovarian cancer compared to controls | n.a | [80] |
| CD59 |  |  |  |  |  |
|  | Head and neck cancer | Cell line  Human tissue | Strong expression of the CD59 cancer cells and in newly derived tumor tissue, metastatic lymph node tissue, tumor infiltration and peripheral blood lymphocytes | n.a | [86] |
|  | Pancreas | Pancreatic cancer Cells lines | Higher expression of CD59 predicts shorter survival for patients with pancreatic cancer | n.a | [81] |
|  | Ovarian carcinoma | Cell line | Cancer cells express high CD55 membrane-bound | n.a | [87] |
|  | Ovarian cancer, corpus uteri cancer | Cell line  Human tissue | Increased expression of CD59 in ….. malignant tissue | n.a | [88] |
|  | Ovary and corpus uteri tumors | Human tissue | Significant expression of CD59 in ovarian cancer tissue | n.a | [80] |
|  | Breast cancer | Breast cells | Retroviral vector-mediated RNAi successfully suppressed the *CD59* gene for breast cancer cells | n.a | [79] |
| Anti-tumor Role | | | | | |
| C1q complex |  |  |  |  |  |
|  | HER-2 breast cancer | Mouse model | C1q ….. the tumor angiogenesis and induces apoptosis in mammary cancer cells by coordinating the signal transduction pathways linked to C1q-WWOX in mammary tumor immune surveillance | C1q-WWOX | [23] |
|  | Prostate | Cell line | complement C1q may induce apoptosis of prostate cancer cells by activating WOX1 and destabilizing cell adhesion. Downregulation of C1q enhances prostate hyperplasia and cancerous formation due to failure of WOX1 activation. | C1q-WWOX | [22] |
| C7 |  |  |  |  |  |
|  | Non-small cell lung cancer | Human tissue | C7 has a tumor suppressor role Low C7 expression was associated with worse outcome as well as with clinical stage and grade | n.a | [75] |
| Properdin |  |  |  |  |  |
|  | Breast cancer | Mouse model | Properdin induces modulation of cell apoptosis of breast cancer cells  Properdin acts as a new tumor suppressor pathway by TES induction and DDIT3 increase | TES-DDIT3 | [92] |

**n.a:** not available; **TAMs:** tumor-associated macrophages; **mRNA:** messenger RNA; **MES:** mesothelioma cells; **MMP9:** Matrix metallopeptidase 9; **DDR1:** Discoidin domain receptors **PSA:** prostate-specific antigen; **VEGF:** Vascular endothelial growth factor; **PDOXL1:** podocalyxin-like protein 1; **LSF-1:** late SV40 factor or *TFCP2* gene **DAF:** decay accelerating factor; **GC:** gastric cancer; **RNAi:** interference RNA; **NSCLC:** Non-small cell lung cancer; **TES:** testin LIM domain protein; **DDIT3:** DNA-Damage-Inducible Transcript 3; **ERK1/2:** extracellular signal-regulated kinases; **SAPK:** stress-activated protein kinases; **JNK:** Jun amino-terminal kinases; **AP:** alternative pathway; **C3aR:** C3a receptor; **C5aR:** C5a receptor; ***FGF1*:** fibroblast growth factor gene; ***RCG32*:** complement response gene 32; **RhoA:** Ras homolog family member A; **RGS16:** regulator of G-protein signaling 16; **IRF1:** interferon regulatory factor 1; **MMP-3:** matrix metalloproteinase-3; **MMP-13:** matrix metalloproteinase-13; **CXCL1:** g chemokine (CXC motif) ligand 1; **EGRF:** epidermal growth factor receptor; **AREG:** amphiregulin; **FH:** factor H; **FHL-1:**  Factor H-like 1; **IL-6:** interleukin 6; ***WWOX:*** WW Domain Containing Oxidoreductase gene.
